# C–H … N hydrogen bonding in an overlayer of s-triazine physisorbed on a graphite surface

**DOI:** 10.1080/00268976.2019.1706777

**Published:** 2019-12-26

**Authors:** Jonathan A. Davidson, Stephen J. Jenkins, Fabrice Gorrec, Stuart M. Clarke

**Affiliations:** a Department of Chemistry, University of Cambridge, Cambridge, UK; b MRC Laboratory of Molecular Biology, Cambridge, UK; c BP Institute, University of Cambridge, Cambridge, UK

**Keywords:** Monolayer, physisorption, diffraction, hydrogen bonding, DFT

## Abstract

The structure of a crystalline monolayer of 1,3,5-triazine has been characterised using X-ray diffraction. The monolayer is found to exhibit a hexagonal unit cell with a lattice parameter of 6.161(5) Å, indicating the formation of C–H … N hydrogen bonds. DFT simulations have been performed exhibiting close agreement with the experimental structure. By comparing the strength of the intermolecular interactions both with and in the absence of Van der Waals corrections, it is possible to estimate an interaction strength for the weak C–H … N hydrogen bonds.

## 1 Introduction

The study of self-assembly of physisorbed systems at surfaces is currently of great interest. These systems consist of molecular species that are weakly physisorbed to the surface of a bulk material while exhibiting stronger non-covalent interactions with themselves and co-adsorbates. Due to the reversible nature of both the adsorbate–surface and adsorbate–adsorbate interactions, these systems are generally observed at thermodynamic equilibrium, unlike many other surface-confined systems. These systems show promise for applications in nano-templating and surface property modification. In addition, they also have the potential for ‘responsive’ behaviour, as the energy required to break the intermolecular bonds is not prohibitive.

**Figure 1:**
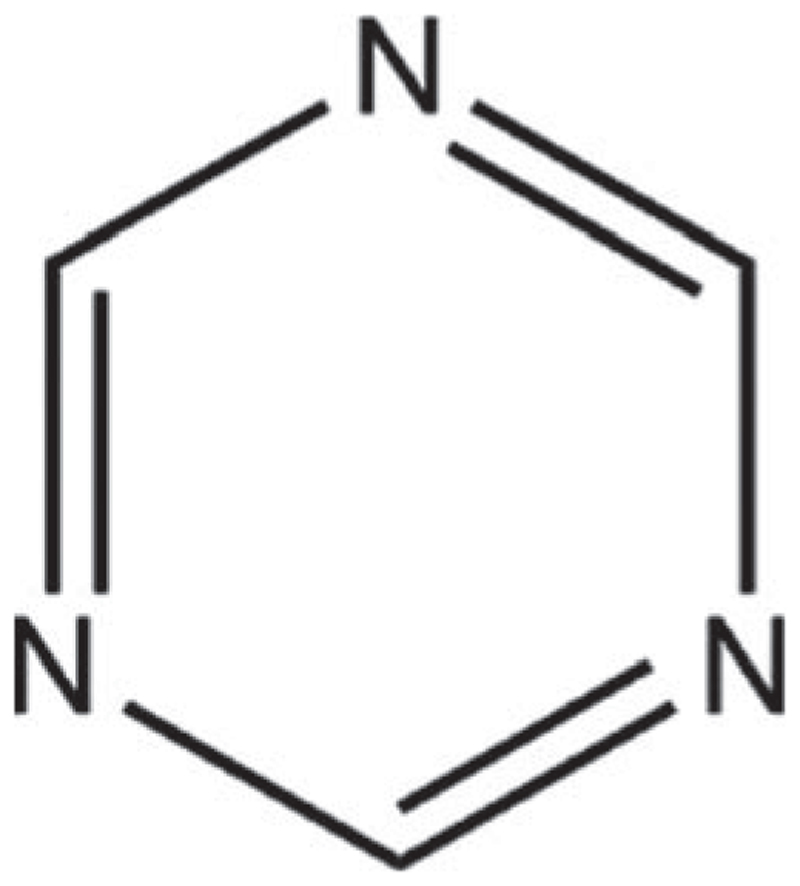
Chemical structure of 1,3,5-triazine.

Due to the negligible amount of material at the surface compared to the bulk, monolayers are comparatively difficult to study. Historically, scanning probe techniques (STM, AFM) have been the preferred method, particularly in ambient conditions. However, complementary techniques that are non-invasive are sought to better understand possible perturbations that can be introduced by the scanning probe [1–3]. Powder X-ray diffraction (XRD) using a high surface area substrate has previously been successfully used to study several halogen bonding systems [4,5]. As well as the potential ligands [6,7]. However, the requirement for synchrotron radiation has limited the ability to fully explore the capabilities of the technique. In this work, we successfully employed a commercially available X-ray diffractometer to collect high-quality data and characterise the formation of solid monolayers of 1,3,5-triazine (Figure 1) on a graphitic surface at ambient pressure to sub-angstrom precision.

1,3,5-triazine its derivatives are important in a range of areas, both as ligands for supramolecular self-assembly of more complex mixed layers, and for their potential to modify the electronic structure of graphene-like materials [8]. Unlike the previously studied halogen bonding systems, this molecule consists only of weakly scattering light atoms and hence provided a challenging test for the more general applicability of our approach.

## 2 Methodology

### 2.1 Experimental

The experimental method used in this work has been detailed elsewhere [5]. The graphite substrate used is Papyex, an exfoliated recompressed graphite foil from Le Carbon. The structure of Papyex is such that the graphite crystallites are highly aligned in the plane of the sheet, allowing manipulation of diffraction geometry to optimise scattering from the in-plane monolayer peaks. The batch of Papyex used in this work was 0.5 mm in thickness, and experimentally determined to have a (BET) surface area of 15.61 m^2^ g^−1^. It was outgassed under vacuum for 6 h at 673 K before dosing. 1,3,5-triazine was obtained from Sigma-Aldrich (97%) and was used without further treatment.

Dosing was performed from the vapour phase. Weighed amounts of graphite and adsorbate were loaded into Pyrex tubes, which were evacuated to a pressure of ca. 0.1 mbar and sealed under vacuum. The tubes were then heated to 423 K, before being left to cool slowly to room temperature to anneal. After cooling, the tubes were opened and the dosed papyex recovered. Dosing was performed such that coverage was approximately 0.8 ML, based on an estimate of the molecular area.

A system comprising a Rigaku FR-E+ superbright (rotating copper anode, 200-*μ*m beam) diffractometer with MAR-DTB image-plate detector at the Laboratory of Molecular Biology (LMB) in Cambridge was used. A graphite monochromator was used to generate X-rays with a wavelength of 1.54179 Å. The sample was cooled to 100 K using a nitrogen cryostream (Oxford Cryostream). Sample geometry was flat-plate transmission, and the detector-sample distance was set to 350 mm, which gave a maximum 2*θ* range of 28°. Calibration of the detector angles was performed using a papyex strip coated in silver behenate. Integration of the obtained powder rings onto a single radial dimension was performed using the fit2D software platform [9,10]. Further analysis of the data was then performed using a custom python script ‘PatternNx’ that accounts for the observed ‘sawtooth’ lineshape of 2D diffraction peaks [5,11].

Bulk diffraction data for comparison purposes was extracted from the Cambridge Structural Database (CSD) [12], with analysis performed using the Mercury software package [13].

### 2.2 Computational

First-principles density functional theory (DFT) calculations were carried out using periodic boundary conditions, as implemented in the CASTEP computer code [14]. In view of the expected lack of any strong interaction between graphite and the adsorbed molecules, the substrate was omitted from the adopted model. Instead, an isolated triazine raft was modelled within a supercell of length 12 Å, with lateral dimensions initially conforming to a lattice constant of 6.15 Å (based upon an early estimate of the experimental value). Atomic positions and the lateral lattice constant were then refined according to the calculated forces, including finite-basis correction, with the lattice angles constrained to hexagonal symmetry. Geometry convergence was gauged with respect to an energy tolerance of 10^−5^ eV, a force tolerance of 0.02 eV.Å^−1^, and a stress tolerance of 0.01 GPa.

The Kohn–Sham wavefunctions of the system were expanded in a plane-wave basis set, up to a kinetic energy cutoff at 600 eV, while the Brillouin zone was sampled over a 3 × 3 × 1 Monkhorst–Pack mesh [15]. Electron-ion interactions were included through the use of ultrasoft pseudopotentials [16] from the standard CASTEP library, and the exchange-correlation interaction was represented by the Perdew–Burke–Ernzerhof (PBE) functional [17]. In order to test the sensitivity of the results to Van der Waals (VdW) interactions, beyond the capacity of standard DFT, two semi-empirical correction schemes were compared, namely Grimme’s D2 scheme [18] and the Tkatchenko–Scheffler (TS) scheme [19].

## 3 Results

### 3.1 Experimental data

**Figure 2:**
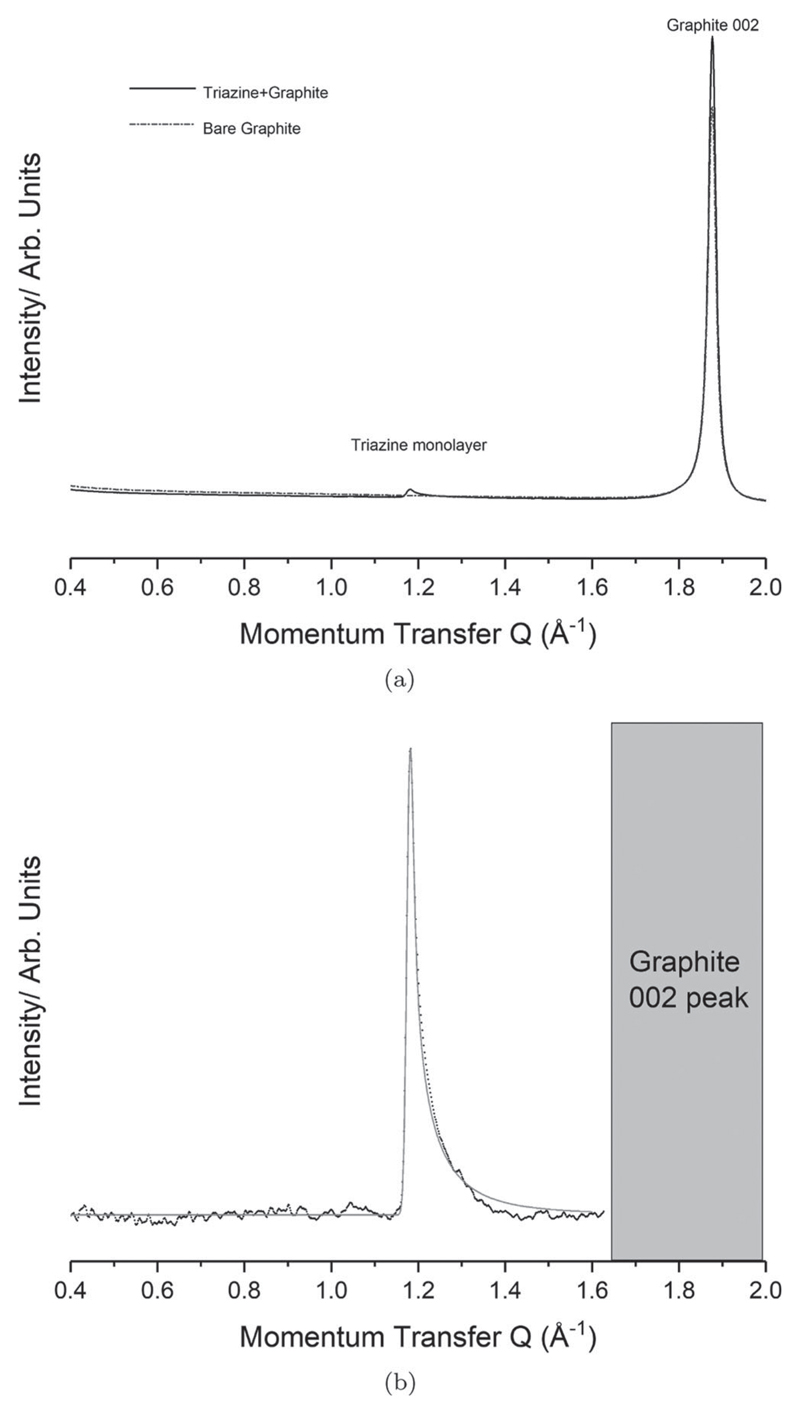
Collected diffraction data for the triazine monolayer system. (a) Experimental diffractogram collected for Dosed (solid) and Bare (dashed) graphite. It is evident the bulk graphite peak is substantially more intense than the monolayer signal. (b) Background subtraction of the monolayer pattern (black) compared to the modelled pattern (grey).

Figure 2(a) presents the collected diffractogram for a bare graphite sample, together with that for a sample dosed with 0.8 ML of s-triazine. The preferred orientation of the papyex and the flat-plate geometry used both favour diffraction by in-plane atomic spacings. However, it is still clear that the out-of-plane (002) peak of the graphite is substantially more intense than the monolayer signal. This relative weakness of the monolayer signal is why diffraction studies of this type can be so challenging. Figure 2(b) then presents the diffraction pattern of the monolayer after subtraction of the graphite background. Incomplete subtraction of the Graphite peak limits the range of high Q peaks that can be reliably observed. Below *Q* = 0.4 small angle ‘Porod’ scattering is evident arising from the dimensions of the graphite crystallites. No significant 2D peaks were observed in this region so it is removed from consideration. A strong peak with ‘sawtooth’ lineshape is observed at *Q* = 1.18 Å^−1^.

When interpreting powder diffraction data it is generally held that the highest symmetry structures should be considered first, with lower symmetry structures considered only if no satisfactory high-symmetry assignment is possible. Hence, this single peak is interpreted as being due to a hexagonal unit mesh, which is consistent with previous STM results [20]. Using this assignment, it is possible to fit this peak to obtain the size of the monolayer lattice parameters. The grey line in Figure 2(b) presents the best fit for the monolayer structure. The lattice size was fit as *a* = *b* = 6.161(5) Å. The coherence length was fit to be 405 Å. It is typical for monolayers of this type to exhibit coherence lengths on the order of magnitude of the coherence length of the substrate (ca. 600 Å) [21]. Previous authors, using the periodicity of moire patterns imaged using STM have reported lattice constants of 6.14 Å at 40 K [22]. This value is approximately 0.3% smaller than that found here, which could be due to thermal contraction.

**Figure 3:**
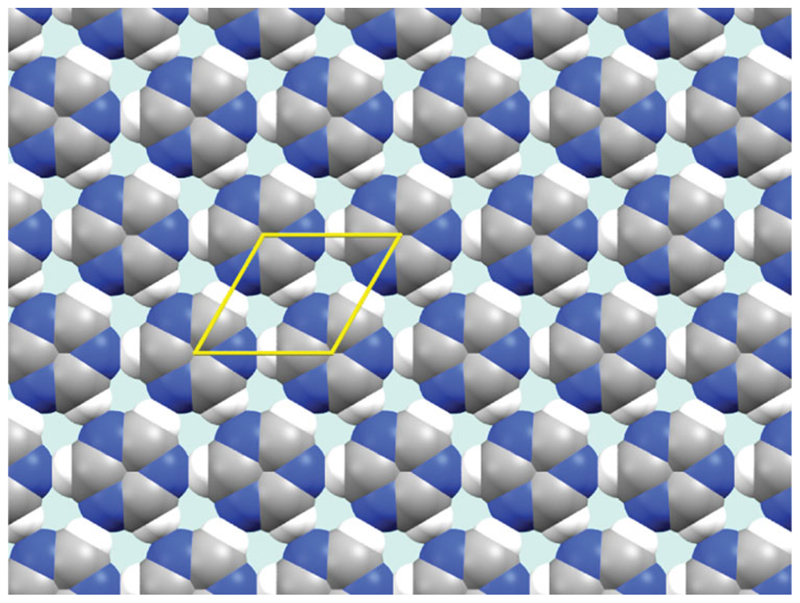
Proposed structure of the triazine monolayer, based upon logical placement of s-triazine molecules onto the experimentally determined lattice.

As there is only one peak in the region of the diffraction pattern observed, it is impossible to assign relative peak intensities, and thus atomic positions within the unit cell. In the past, the monolayer structure has been generated by assuming that molecular structures are unchanged from the single crystal structure deposited in the CSD. These molecular structures can then be placed on the experimentally determined lattice. However, as this molecule contains many light atoms (in particular hydrogen) X-ray structures may not provide the most accurate structure for calculation of intermolecular contacts. A literature molecular structure derived from electron diffraction data [23] has previously been benchmarked against DFT simulations [24]. This structure is similar to that listed in the CSD, however, has significantly different C–H bond lengths. The monolayer structure is shown in Figure 3.

**Figure 4:**
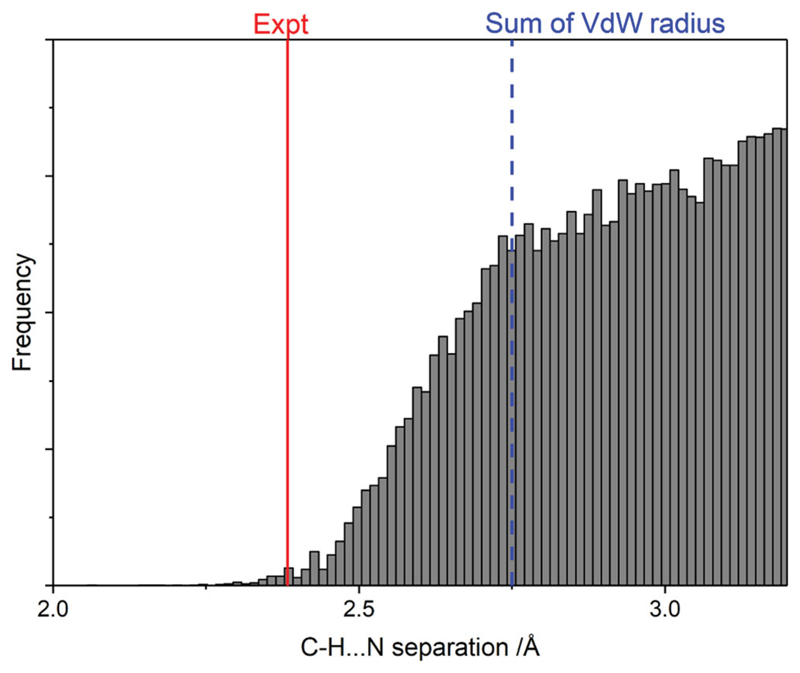
Frequency of various C–H … N distances in the CSD. The distance of the proposed structure is highlighted, and is found to fall within a reasonable range for a hydrogen bonded system.

This structure exhibits a linear H–N bond distance of 2.383(3) Å. In the three-dimensional bulk structure the H-N separation is not linear and is comparatively large at 2.799 Å. For comparison purposes, Figure 4 shows the frequency distribution of various C–H … N distances found in the CSD for aromatic protons interacting with aromatic nitrogen-containing rings. The experimental monolayer distance is significantly shorter than most previously reported distances, and is ∼ 14% lower than the combined VdW radii of the two elements. This suggests a genuine hydrogen bond.

### 3.2 Simulation

Initial work was performed to validate the experimental lattice constant. In the absence of semi-empirical correction for VdW interactions, we found a lattice constant of 6.15 Å, which is 0.2% smaller than the experimental value. Inclusion of either VdW correction scheme resulted in an optimised lattice constant that was consistently lower, but still only marginally below that found in our experiments: 6.08 Å (−1.3%) using the D2 correction, and 6.07 Å (−1.5%) using the TS correction. In all three cases, the molecular structures were essentially identical, with C–N bond lengths of 1.33 Å and C–H bond lengths of 1.09 Å. Intermolecular H/N distances were therefore 2.39 Å in the calculation without semiempirical corrections, but 2.31 Å with the D2 correction and 2.33 Å with the TS correction. Corresponding intermolecular C/N distances were 3.48 Å without correction, 3.42 Å with the D2 correction, and 3.40 Å with the TS correction.

The above distances are even shorter than those estimated in the experimental data, and are very short compared to the typical C–H … N separation shown in Figure 4. When considering to what extent the interactions can be characterised as C–H … N hydrogen bonds, it is significant that all three calculations indicate a substantial electron-withdrawing influence from the nitrogen atoms upon the carbon atoms. Hirshfeld analysis [25] yields charges of −0.13*e* on the nitrogen atoms, +0.09*e* on the carbon atoms, and +0.04*e* on the hydrogen atoms; results of a Mulliken analysis [26] are even more dramatic, with charges of −0.44*e* on nitrogen atoms, +0.08*e* on carbon atoms, and +0.36*e* on hydrogen atoms, but are probably less physically reasonable.

It is possible to estimate the strength of intermolecular interactions by comparing the energy change in going from the bound system to one with double the lattice parameter and hence (nearly) no intermolecular interaction. If this is performed without VdW corrections, it is found that intermolecular interactions combine to stabilise the raft by 0.27 eV per molecule (0.09 eV per putative hydrogen bond). With the D2 correction, the corresponding stabilisation is 0.40 eV per molecule (0.13 eV per hydrogen bond) while with the TS correction we obtain 0.41 eV per molecule (0.14 eV per hydrogen bond). Since the attractive interaction appears not to be predominantly derived from the VdW components, we tend towards the view that weak C–H … N hydrogen bonds are indeed formed in this system.

## 4 Conclusions

Using a combination of experimental and theoretical techniques, the structure of a solid monolayer of triazine on graphite has been probed to a greater degree of precision than has previously been possible. This demonstrates the importance of using a range of experimental techniques when studying such systems. High-quality DFT simulations have also allowed further exploration of the nature of the intermolecular interaction in the layer. It is found that the interaction has a significant electronic component, indicating the system can be considered to exhibit hydrogen bonding. As well as the requirement for high-quality experimental data for benchmarking of theoretical studies, it is hoped availability of high flux lab-based instrumentation will allow greater utilisation of scattering techniques for the study of surface-based systems.
